# Health and tuberculosis systems resilience, the role of the private sector and pandemic preparedness: insights from a cross-country qualitative study with policy-makers in India, Indonesia and Nigeria

**DOI:** 10.1136/bmjgh-2024-016180

**Published:** 2025-01-20

**Authors:** Laura Jane Brubacher, Vijayashree Yellappa, Bony Wiem Lestari, Petra Heitkamp, Nathaly Aguilera Vasquez, Angelina Sassi, Bolanle Olusola-Faleye, Poshan Thapa, Joel Shyam Klinton, Surbhi Sheokand, Madhukar Pai, Charity Oga-Omenka

**Affiliations:** 1 School of Public Health Sciences, University of Waterloo, Waterloo, Ontario, Canada; 2 TB PPM Learning Network, McGill International TB Centre, McGill University, Montreal, Québec, Canada; 3 Research Center for Care and Control of Infectious Disease, Universitas Padjadjaran, Bandung, Indonesia; 4 Department of Public Health, Faculty of Medicine, Universitas Padjadjaran, Bandung, Indonesia; 5 Research Institute of the McGill University Health Centre, McGill University, Montreal, Québec, Canada; 6 Sustaining Health Outcomes through the Private Sector (SHOPS) Plus/Abt Associates, Lagos, Nigeria

**Keywords:** Global Health, Health services research, Health systems, Tuberculosis, Qualitative study

## Abstract

**Introduction:**

The COVID-19 pandemic was an unprecedented challenge to health systems worldwide and had a severe impact on tuberculosis (TB) case notifications and service delivery. India, Indonesia and Nigeria are high TB-burden countries where the majority of initial care-seeking happens in the private health sector. The objectives of this study were to (1) explore policy-makers’ perspectives on the impact of the COVID-19 pandemic on private sector TB service delivery in India, Indonesia and Nigeria and (2) identify cross-cutting insights for pandemic preparedness with respect to TB service delivery.

**Methods:**

From May to November 2021, 33 interviews were conducted with key policy-makers involved in health service administration, TB service delivery and/or the COVID-19 response in India, Indonesia and Nigeria (n=11 in each country). Interviews focused on the impact of COVID-19 on TB services and lessons learnt for pandemic preparedness with respect to TB in each study context. Data were analysed thematically using a hybrid inductive-deductive approach, informed by Haldane *et al*’s Determinants of Health Systems Resilience Framework.

**Results:**

Policy-makers highlighted the crucial role of intersectoral collaboration, effective governance, innovative financing strategies, health workforce reallocation and technological advancements such as virtual consultations and mHealth in strengthening TB service delivery amid the COVID-19 pandemic. India relied on patient–provider support agencies to implement a joint strategy for TB care across sectors and states. Indonesia engaged networks of private provider professional associations to facilitate coordination of the COVID-19 response. Nigeria implemented a pandemic policy for public–private referral for the continuity of TB care.

**Conclusions:**

Countries implemented varied measures to support TB service delivery during the COVID-19 pandemic. This study presents insights from three countries (India, Indonesia and Nigeria) that together offer a ‘menu’ of possibilities for supporting pandemic preparedness with respect to TB care vis-à-vis strengthening health systems resilience.

**WHAT IS ALREADY KNOWN ON THIS TOPIC**

**WHAT THIS STUDY ADDS**

- All three countries leveraged private sector professional associations’ networks and NGOs to support a coordinated approach to delivering TB services and controlling the transmission of COVID-19 across sectors. The private sector of all countries helped carry the burden of TB care when the public sector was overwhelmed by COVID-19; however, countries varied in their use of other strategies to support public health functions, health workforce distribution and health service delivery.

**HOW THIS STUDY MIGHT AFFECT RESEARCH, PRACTICE OR POLICY**

- This study underscores the importance of a collaborative, community-engaged approach as we strive for more effective and adaptable TB care in the face of future health crises.

## Introduction

 COVID-19 and tuberculosis (TB) are the two deadliest infectious diseases globally, with 6.7 million and 4.2 million deaths from COVID-19 and TB between 2020 and 2022, respectively.^1 2^ Both are respiratory tract infections with some similarities in transmission, symptoms and public health strategies.^3 4^ Since the pandemic, public health responses to TB have been severely impacted globally, as countries prioritised their COVID-19 responses over other public health functions.^1 3^

The WHO Global TB Reports since 2020 highlight the complex impact of the COVID-19 pandemic on TB^5 6^ and TB care.^1^ TB case notifications—a critical component of disease surveillance and pandemic preparedness—dropped globally in 2021, from 7.1 to 5.8 million cases between 2019 and 2020, with some increase to 6.4 in 2021 and full recovery to 7.5 million in 2022. These drops were observed in most high-burden countries such as India, Indonesia and the Philippines, with a few countries in the African region showing some increases in notifications between 2019 and 2022. These reports indicate significant challenges in accessing TB care during the pandemic, with less impact in a few countries.

### Research locations: India, Indonesia and Nigeria

India, Indonesia and Nigeria are high TB burden countries, with the highest proportions of initial care-seeking taking place in the private sector and with different trends in TB notifications since the pandemic.^1^ By mid-2021, when this study was conducted, these three high TB burden countries showed markedly different trajectories in their TB service recovery from COVID-19’s initial disruptions in 2020, which included case notification decreases of 25% in India and 31% in Indonesia, and an increase of 15% in Nigeria, following initial disruptions.^7^ Indeed, while India and Indonesia were grappling in mid-2021 with devastating new COVID-19 waves that repeatedly strained their health systems, Nigeria had already achieved full recovery of TB notifications and was continuing its pre-pandemic improvement in case detection, rising from 24% in 2017 to 45% by study completion. While invariably shaped by the differing intensity of COVID-19 waves, these trends in service recovery may also signal some differences in TB case finding and health system responses within each country. Diverse COVID-19 control measures implemented by different countries might have differentially impacted TB care. For example, countries such as Nigeria that experienced previous outbreaks of infectious diseases like Ebola may have leveraged these experiences to inform their pandemic responses.^8^,^10^

### Health system structures and public–private mix initiatives

These countries’ health systems share a complex mix of public and predominantly private for-profit healthcare delivery, though vary in their private healthcare configurations.^11^ India’s private sector is notably pluralistic, encompassing multiple systems of medicine (allopathic, ayurveda, unani, Siddha and homoeopathy), ranging from corporate hospitals and non-profit trusts to individual practitioners providing primary care in both rural and urban settings, and accounting for about 75% of initial care-seeking.^11 12^ Indonesia’s private sector includes over 24 700 pharmacies, 8600 drug shops and 1500 hospitals and accounts for 74% of initial TB care-seeking and 42% of treatment.^11 13^ Nigeria’s private sector, while poorly documented, handles 66%–92% of initial care-seeking for respiratory conditions, particularly through over 60 000 patent medicine vendors.^11^

In all three countries, public–private mix (PPM) initiatives aim to leverage collaboration across public and private healthcare sectors to improve TB case notification. Prior to COVID-19, PPM initiatives revealed both promise and persistent challenges, with all three countries recording limited gains in notifications.^11^ In India, formal PPM initiatives dramatically increased private sector TB notifications from 3533 cases (0% of the country’s total notifications and 0% of estimated incidence) in 2012 to 383 784 cases (21% and 14%) by 2017, with treatment success rates comparable to the public sector at 87%. Indonesia’s efforts to engage private providers through professional societies showed some progress with private notifications rising from 5432 (2% of total notifications and 1% of estimated incidence) in 2012 to 59 549 (13% and 7% of incidence) in 2017. Nigeria’s PPM initiatives, despite existing since 2003 and having clear guidelines since 2006, received little funding and policy backing, with private notifications actually declining from 8121 cases (9% of total notifications and 2% of estimated incidence) in 2015 to 4968 cases (5% of total notifications and 1% of estimated incidence) in 2017.^14 15^ Common obstacles across countries to enhancing PPM initiatives included fragmented health information systems, complex notification procedures, limited private sector capacity for TB management and insufficient financial incentives for collaboration—challenges that would later prove critical during COVID-19 response.

### COVID-19 and private sector engagement

Studies have focused on the impact of COVID-19 on private sector care, specifically the varied responses and adaptations by providers.^16^,^19^ Indeed, while COVID-19 impacted TB services globally through disruptions to private healthcare provision and reduced patient turnout, the private healthcare sector also showed resilience and adaptability in continuing essential TB services despite challenges.^20^ Providers promptly adjusted services, implemented infection control measures and maintained TB care quality.^21^ Understanding private sector TB service delivery adaptations in India, Indonesia and Nigeria during COVID-19 is crucial for pandemic preparedness given that these high-burden countries rely predominantly on private providers for initial TB care-seeking. By analysing their diverse policy responses and innovations, this study provides essential evidence for maintaining TB and other health services in similar settings during future pandemics. This emphasis also aligns with recent calls for enhanced private sector engagement and integration of health systems. In 2023, United Nations High Level Meetings on TB (UNHLM),^22^ Universal Health Coverage (UHC) and pandemic preparedness emphasised the importance of building resilient integrated health systems. WHO is actively positioning TB care within a multisectoral framework^23 24^ and emphasises the importance of private sector engagement in UHC^25^ as well as the Pandemic Accord and related governance systems.^26^

Overall, countries adapted differently to the health system challenges presented through the COVID-19 pandemic; however, there is an opportunity to further understand these adaptations, with an eye to strengthening ongoing private–public collaboration for TB service delivery for future pandemics. The objectives of this study were to (1) explore policy-makers’ perspectives on the impact of the COVID-19 pandemic on private sector TB service delivery in India, Indonesia and Nigeria and (2) identify cross-cutting insights for pandemic preparedness and public health planning with respect to TB service delivery.

## Methods

### Study context

This qualitative study is part of a broader project, the COVID-19 Effect on TB in the Private Sector (COVET) study, which aimed to assess the post-COVID-19 landscape of private healthcare sectors in high TB burden countries that have extensive private sector delivery of TB services: India, Indonesia and Nigeria. The COVET study is a joint research project conducted by the McGill International TB Centre, Georgetown University, University of Waterloo, Universitas Padjadjaran and the USAID-funded Sustaining Health Outcomes through the Private Sector (SHOPS) Plus programme. In India, the Institute of Socio-Economic Research on Development and Democracy and its partners leveraged the Patient Provider Support Agency (PPSA) network for data collection. The PPSA is a health service delivery model in India that involves contracting a third-party agency (ie, non-governmental organization [NGO]) to engage private sector providers in TB service provision. In Indonesia, the Research Center for Care and Control of Infectious Disease-Universitas Padjadjaran collaborated with the local health office to use the study area of the 2019 Investigation of Services Delivered for TB by External Care Systems—Especially the Private Sector study in Bandung.^27^ In Nigeria, the SHOPS Plus programme facilitated partnerships for the study in Kano and Lagos.^28^ This broader project provided a robust methodological context to examine the post-COVID-19 private sector TB landscape across diverse settings.

### Data collection

From May to November 2021, key informant interviews were conducted with policy-makers involved in private sector TB care in India, Indonesia and Nigeria, recruited through COVET study networks and partnerships (n=33 policy-makers; n=11 interviews per country). Interview participants were sampled purposively according to their roles and responsibilities within government TB programmes, communicable disease programmes, general health service administration, private sector professional associations and NGO programme administration and selected as a result of a stakeholder mapping exercise conducted by the research team in each country (table 1). Interviews included policy-makers’ perspectives on the impact of the COVID-19 pandemic on TB health service delivery, the performance of their pandemic responses and lessons learnt from the impact of COVID-19 on TB services in their respective countries (see online supplemental file 1 for a sample interview guide). Interviews were conducted virtually in adherence to COVID-19 safety guidelines and in English, except for one interview each in Hindi (India) and Bahasa (Indonesia), as per the participants’ preferences. Interviews in Hindi and Bahasa were subsequently translated and transcribed into English. Interviews were approximately 50 min in duration (range: 30–60 min) and were audio-recorded with permission. All individuals provided verbal informed consent to participate.

**Table 1:** Key informant interview participants' organisational roles or affiliations

Organisational role and level (when applicable)	Participant ID*
TB programme administration†	
National (n=3)	I1, D2, N9
State/provincial (n=6)	I2, I4, I5, D4, N2, N5
District/city (n=1)	I3
Health service administration†	
State/provincial (n=1)	D5
District/city (n=3)	D3, D10, D11
Communicable disease control programme administration†	
State/provincial (n=4)	N1, N3, N6, N7
District/city (n=1)	D1
Private sector professional association or monitoring agency‡	
State/provincial (n=4)	D6, D7, N4, N8
District/city (n=1)	D9
Non-governmental organization programme administration (n=8)	I6, I7, I9, I10, I11, D8, N10, N11
Academic/researcher (n=1)	I8

* I=India participant; D=Indonesia participant; N=Nigeria participant.

† Roles within the public, government health sector.

‡ Roles within or adjacent to the private health sector.

TBtuberculosis

### Patient and public involvement

Patients or the public were not involved in the design, conduct, reporting or dissemination plans of this research.

### Data analysis

Data were analysed thematically, using a constant comparative approach within and across interview transcripts, to identify insights into COVID-19 impacts and adaptations.^29^ A hybrid inductive-deductive coding approach was employed.^30^ Research teams in each country conducted an initial inductive thematic analysis. Subsequently, teams met to compare and contrast their coding and develop analytic insights across countries. This analysis created the foundation for subsequent secondary analysis by the University of Waterloo team (LJB and CO-O)—with training in public health, health systems and services research, and qualitative methodology—to map inductive codes to Haldane *et al*’s Determinants of Health Systems Resilience Framework.^31^

The Haldane *et al*’s framework builds from the WHO’s health systems ‘building blocks’^32^ to include public health functions—critical to pandemic response—and conceptualises elements of health systems that promote capacity to ‘prepare for, recover from and absorb shocks, while maintaining core functions’ (32:964). Central to this framework is the notion of community engagement as integral to the building of systems-level resilience. This framework was particularly appropriate for examining country responses to the COVID-19 pandemic (a notable systems ‘shock’) and understanding the capacity of systems to maintain essential TB care functions (ie, testing and treatment) amid this shock. Mapping our inductive codes on countries’ responses to the structure of this framework more readily facilitated comparative analysis across countries. We adapted the framework’s cross-cutting domain of intersectoral collaboration to ‘public–private health sector engagement’ to increase its relevance to TB care, whereby PPM initiatives are a significant locus of collaboration and engagement. Future studies that aim to understand TB systems resilience amid shocks (eg, pandemics) may benefit from a similar adaptation. NVivo V.14 software was used for organising and retrieving codes and coded excerpts. Collaboration among the research team and peer debriefing contributed to the validity of the analysis.^33^

## Results

Policy-makers’ perspectives on the impact of the COVID-19 pandemic on private TB services and adaptations mapped to the following core domains of the health systems resilience framework^31^: governance and financing; health workforce and health service delivery; public health functions and medical products and technologies. Importantly, results also highlight increased public–private health sector engagement across countries’ health system responses to COVID-19.

### Increased public–private health sector engagement

Across countries, policy-makers underscored how COVID-19 amplified public–private sector engagement, with implications for TB care moving forward. In Indonesia, a participant from the private sector noted the reliance of the public sector on private clinics to ‘roll out’ the COVID-19 vaccination programme–that it was beneficial that the public sector had to determine how to use and collaborate with private clinics: ‘*In the beginning, they heed[ed] no attention towards clinics, but eventually they asked for our help. And this whole thing changes the paradigm—after all, we are present not as competitors’* (D6). Similarly, Nigerian policy-makers reported an ‘all hands-on deck’ public–private health sector collaboration evident in the pandemic that can be leveraged into TB service delivery (N9). This built on a strong pre-existing role of the private sector in TB care, which provided a ‘cushion’ to fill gaps when public sector care was difficult to access during pandemic lockdowns (N5). In India, PPSAs actively collaborated during the pandemic with the National TB Elimination Programme (NTEP) to develop and implement a joint strategy for TB service delivery across states and sectors (I1). Beyond case notifications, one policy-maker in India also described how COVID-19 opened and improved existing lines of communication between sectors (vis-à-vis a Deputy Commissioner’s communication with the private sector regarding district COVID-19 transmission), which may support TB service delivery in the future (I3). Based on the experience of private–public health sector collaboration in COVID-19, a policy-maker in India also described what an increased PPM could look like moving forward, with relevance to TB service delivery:

Ultimately, the heads of the organizations either in the public or private sector, there will be a face – whoever it is. For example, it’s the director of health services and there would be a similar representative from the private sector. I think that level of conversation of the local IMA [Indian Medical Association] with government was very critical for information to percolate. Unless the top people are convinced, it would not go down. I think those meetings with heads of organizations and then percolating it and cascading it down to their respective members and units was probably the best way (I7).

Overall, there was evidence of the crucial role played by the private sector in TB care during COVID-19 and an identified need for continued strengthening of public–private health sector engagement for the purpose of (a) augmenting the public system and filling human resources gaps in TB service delivery through the private sector vis-à-vis reallocation of the health workforce, extending the ‘reach’ of care and extending the capacity of the health system overall; (b) dissemination of guidelines, policy and training for consistency and clarity of approaches to TB care amid a pandemic, across public–private health sectors and (c) expanded distribution networks for personal protective equipment (PPE), TB medications and vaccines through private facilities (described in detail below). This importance of private health sector engagement in TB care, as illustrated in the COVID-19 pandemic, is integrated into and elaborated on in the following sections—which map to the core domains of Haldane *et al*’s Determinants of Health Systems Resilience Framework.^31^

### Governance and financing

The crucial role of private provider professional associations or NGOs as intermediaries between the public sector and a network of private facilities (ie, PPSAs in India) was highlighted across all three countries. In Indonesia, standardising COVID-19 protocols (ie, PPE usage) and overall coordination and communication across facilities occurred via associations like ASKLIN (private clinic association). While these associations were seen as ‘nodes’ through which increasing public–private engagement was observed in COVID-19 (D6), respondents also felt the public sector predominantly sets standards for private clinics. For instance, PPE requirements were set without any government support for private providers, which increased their operational expenses (D5). A policy-maker noted the need to incentivise the private sector (through finances, infrastructure and tools) to implement government programmes, highlighted in the pandemic and relevant to TB services (D4). Similarly, in Nigeria, the networks of professional associations were leveraged to disseminate policies and practice guidelines from the public sector and distribute PPE. A central government agency (Health Facility Monitoring and Accreditation Agency), which provides performance monitoring and accreditation to private facilities, was also leveraged to disseminate COVID-19 infection prevention and control protocols through its private provider networks. Nigeria also established a more formalised structure for integrated TB and COVID-19 response across public and private sectors vis-à-vis a ‘State TB-COVID-19 response team’ (Kano State). In India, policy-makers identified a need for more foresight as to how to leverage the resources and capacity of the private sector as well as a general need for enhanced partnership and lines of communication across sectors rather than unilateral communication (ie, from the NTEP to private providers). This was underscored in the COVID-19 pandemic but has relevance to TB.

Overall, as stated by a policy-maker in India, there was a political commitment to a multisectoral, community-engaged and collaborative approach to the COVID-19 pandemic response, which could be leveraged and replicated to support progress in TB service delivery:

Things will not happen in the field just by issuing letters and guidelines and unless we sit with the states and be a part of their implementation, it is not going to happen. The technical support agencies, the PPSAs, the civil society partners, the community partners, a lot of them, all of them joined hands to give a response to COVID. And if we can do that, all of government approach for COVID, then we can do that for TB as well. That is what we are going forward with. And there is absolutely full commitment from the administrative setup, from the political setup and from the implementers. All the three are trying to go in one direction (I1)

### Health workforce and health service delivery

Policy-makers across countries identified the need for reallocation of health human resources amid the pandemic, to cover service gaps due to staff shortages and ensure continuity of care for other diseases, like TB. A Nigerian policy-maker observed that this *‘task shifting’* of healthcare workers occurred more readily within the private sector than the public whereby *‘sometimes there’s a vacuum or disruption in service’*, as was observed during the pandemic when health human resources were directed towards COVID-19 prevention and treatment (N11). In Indonesia, a centralised system of mapping and distributing the health workforce across the public system existed, whereby there was coordination between public health facilities, especially at the primary care level like Puskesmas (Pusat Kesehatan Masyarakat), to cover gaps (D3). A policy-maker in India identified how training private sector doctors and district TB officers, alongside creating awareness to the public about the availability of TB diagnosis in COVID-19 in government facilities, has increased case notifications (I4).

Countries employed a variety of strategies, engaging different organisational ‘actors’ to facilitate treatment continuity for TB patients amid COVID-19. In India, the public NTEP implemented a large-scale telephone follow-up and TB treatment intervention to ensure door-to-door delivery of treatment (I4) as well as over-the-phone treatment adherence support through call centres (I6). Health sector NGOs and PPSAs communicated with patients regarding the availability of TB medication and actively sourced public sector drugs for patients unable to access private sector drugs in COVID-19 (I5, I7). Nigeria had a policy that allowed referral of TB patients from the public sector to nearby private hospitals for continuity of care (N10); longer prescription refills given across private and public facilities to avoid unnecessary visits (N2); home-based care of patients (N6) and, overall, less of a *‘dividing line’* between public and private sectors to ensure treatment continuity. A Tuberculosis and Leprosy Supervisor within the NTP followed up with TB patients to support treatment adherence irrespective of whether patients accessed care at private or public health facilities (N11). In Indonesia, private clinics often refer TB patients to public sector facilities (ie, Puskesmas). Policy-makers emphasised that while the pandemic increased overall communication between private and public sectors, it also underscored the need for enhanced reporting mechanisms and TB data synchronisation to reduce patient loss to follow-up (D6, D8).

### Public health functions

Across countries, the pandemic enhanced the capacity of the health workforce to conduct contact tracing for COVID-19 and, consequently, active case finding for TB. One policy-maker in India noted improved *‘overall health system capacity to deal with communicable disease*’ (ie, through staff skills in outbreak management and resources) and that new molecular diagnostic technologies were purchased for COVID-19 which could be leveraged to support TB diagnostics (I1, I2). Similarly, in Nigeria, the pandemic reiterated the importance of active case finding and testing—for COVID-19 and TB—alongside increased public health awareness of these diseases’ similar symptomatology. To do so, Nigeria engaged multiple actors (ie, SHOPS Plus) in community education and health promotion (N2).

The COVID-19 pandemic enhanced monitoring and reporting mechanisms, as shared by policy-makers in India and Nigeria. In India, new mechanisms were established for virtual information exchange and reporting for TB, such as WhatsApp groups among private providers for addressing questions related to TB and COVID-19, as well as mobile applications designed for TB reporting by PPSAs (I10). Nigerian policy-makers noted their already well-established monitoring and evaluation infrastructure (ie, an integrated data surveillance system) within the TB programme at the local government level and across the private and public sectors, which can continue to be leveraged for both TB and pandemic response. Also, in light of the pandemic, TB monitoring and evaluation staff now have the capacity to work virtually, which may confer longer-term benefits in terms of workplace capacity and efficiency (N3, N11).

### Medical products and technologies

Telemedicine technologies were implemented or more effectively used amid COVID-19 across countries. Public sector platforms included e-Sanjeevini in India, Halodoc in Indonesia and EkoTelemed in Nigeria (maintenance or continuation of these technologies at the time of publication was not researched). Private sector facilities developed their own mobile applications for virtual consultations. Mobile applications were also used in both the public and private health sectors for support with TB prescription renewal and treatment continuity. In India, COVID-19 emphasised a need to invest in, mobilise and scale-up information technology infrastructure to facilitate TB treatment adherence in a pandemic (eg, through video-based directly observed treatment). This required training for providers and patients in the use of these technologies. A policy-maker suggested that teleconsultations could be implemented through PPSAs, which are already involved in ensuring TB treatment adherence (I5). Similarly, Indonesian policy-makers identified a need for regulatory change regarding telemedicine or online consultations being recognised as ‘visits’ to facilitate funding to private facilities (D4).

**Figure 1:**
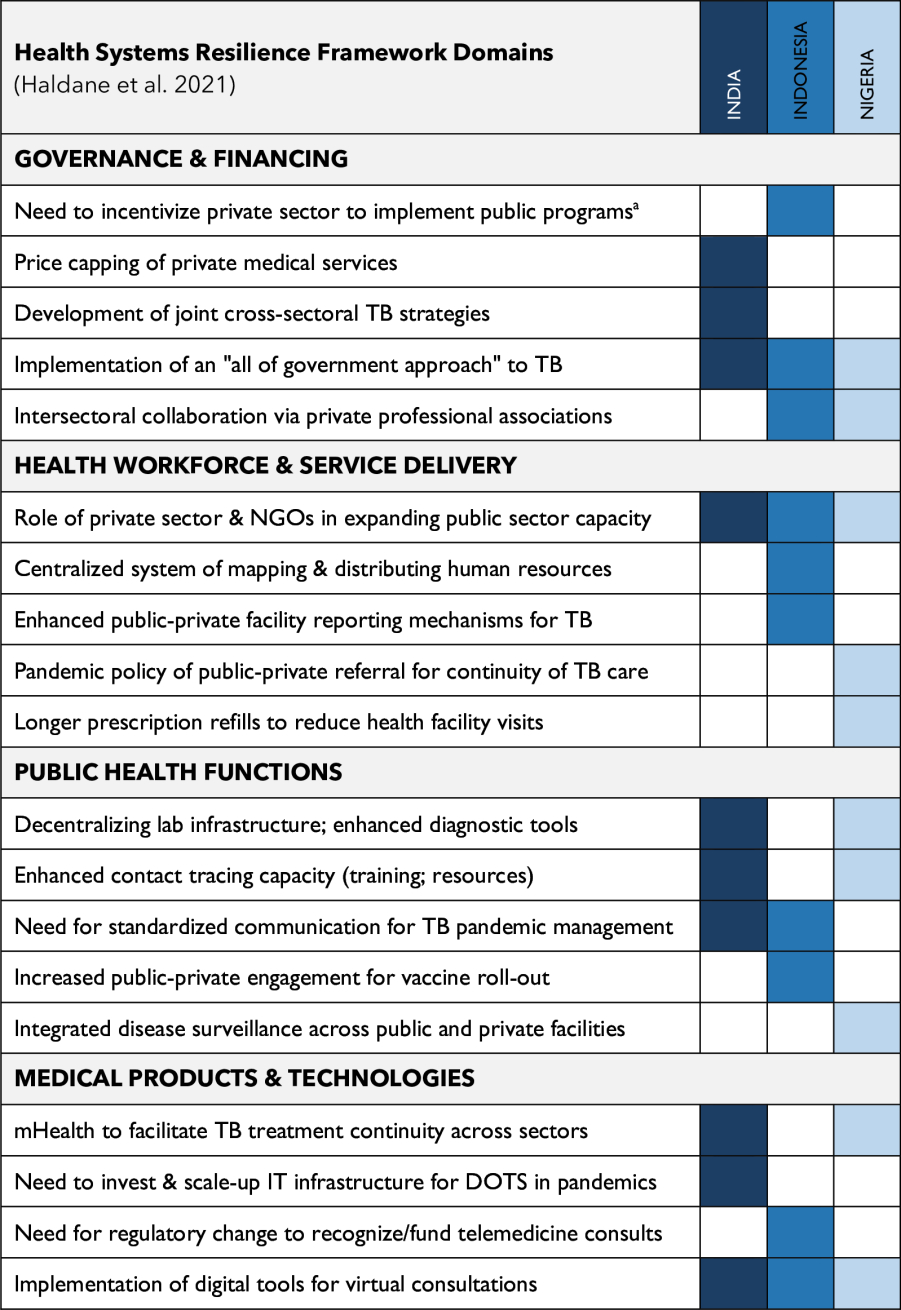
Insights for health systems resilience and pandemic preparedness for TB care. TB, tuberculosis. ^a^Statements beginning with ‘Need to/Need for’ indicate that a participant specified something that was not done or not done sufficiently within their respective country context and is representative of an insight for future pandemic preparedness.

Overall, each country shared evidence of intentional efforts and strategies for coordinating TB care within the COVID-19 pandemic, as well as opportunities for enhancing this coordination for future pandemic preparedness. For example, policy-makers described how the public sector in India relied on private professional associations to implement training for private healthcare workers on TB and COVID-19 infection prevention and control measures (I1). Indonesia strengthened their TB lab network amid COVID-19 and disseminated a circular letter that regulated hospitalisation of drug-resistant TB during the pandemic (D11). Nigeria created a state-level formal structure (within Kano State) for integration of the TB and COVID-19 response, referred to as the State TB-COVID-19 response team (N5). Policy-makers across countries still also identified a need for more consistent and coordinated management of TB within a pandemic setting, in the form of a national-level protocol for TB management in a pandemic (N9); enhanced public–private regulation for TB management (D11) or more intentional leveraging of resources from a substantive private sector to support standardised COVID-19 protocols across public and private health facilities (I10). Actions taken for COVID-19 and TB management that were identified as advantageous by key policy-makers across the three countries mapped closely to Haldane *et al*’s (2021) framework (with health workforce and health service delivery considered together) (figure 1).

## Discussion

As our findings indicate, the private sector in India, Indonesia and Nigeria galvanised in the COVID-19 pandemic to provide TB services. During the pandemic, when public health systems were in disarray, the private health sector mobilised to fill crucial gaps in TB service delivery and to augment the public sector workforce, as well as public health functions (ie, testing, active case finding). The private sector was systematically engaged through various policy and regulatory approaches. Indeed, the strengthening of the private sector that occurred during the pandemic has contributed to current progress in public–private partnerships.^21 34^ These findings align with recent studies in contexts with mixed health systems that similarly underscore the pivotal role of the private sector in providing care for TB patients during a time of uncertainty in which public facilities were overwhelmed.^16 18^

While our findings reveal differing pandemic control and preparedness strategies to varying degrees across countries, we found three major themes across the three countries. These include the implementation of an ‘all of the government approach’ to TB as part of governance and financing; the role of the private sector and NGOs in expanding public sector capacity, particularly for the health workforce and service delivery; and the implementation of digital tools for virtual consultations, as part of TB technologies. These findings align with studies from other countries, including high TB burden countries. Studies have underscored the significance of a robust national strategy in combating the pandemic and how this shows promise for improved TB management and enhanced preparedness for future pandemics.^31 35 36^ The role of the private sector in maintaining services for TB during the pandemic and the increases—although short-lived in most settings—are well documented.^5 17 21 37 38^ Our findings also point to the mechanisms (or ‘nodes’) through which public–private health sector engagement for TB care was occurring during the COVID-19 pandemic across study countries. The public sector interfaced with private providers’ professional associations in Indonesia, as well as with the Private Health Institutions Management Board in Nigeria and PPSAs in India. Opportunities may exist to focus resources on these existing networks, leveraging these partnerships, to enhance TB services.^11 20 21^ NGOs also held a pivotal role in ensuring continuity of TB care in our study, suggesting that NGOs need to be systematically involved both in countries’ NTPs as well as their broader health systems to strengthen resilience.

Several authors have synthesised global data on health systems resilience and pandemic preparedness during various epidemics and highlighted the need for global coordination; guidance, collaboration and information sharing; strengthening weak primary healthcare; acting on social determinants of health; and early, rapid and aggressive actions in public health interventions.^3139^,^43^ Similarly, the WHO Preparedness and Resilience for Emerging Threats initiative calls for an integrated approach to pandemic response, health systems strengthening, enhancing national capacities for planning, coordination, risk communication, community engagement, health intelligence and interventions, as well as global collaboration and expert consultations.^44^ In our study, various features of countries’ overarching governance approaches to the COVID-19 pandemic were highlighted as being relevant to TB delivery moving forward. This includes, most notably, the espousing of a multisectoral and collaborative ‘all of government approach’ to COVID-19 that could be leveraged into TB. This multisectoral, broadly cooperative approach, evident particularly in the early stages of the global COVID-19 pandemic response, has been recognised within the health systems resilience and pandemic preparedness literature as a governance element facilitative of improved outcomes.^31 39 45 46^

Importantly, with respect to TB service delivery, there will not be one approach that fits every context. Rather, our study presents insights from three countries (India, Indonesia and Nigeria) based on actions taken in the COVID-19 pandemic—some approaches were cross-cutting, and others were unique to one country’s context. Together, these findings offer a ‘menu’ of possibilities for supporting pandemic preparedness with respect to TB care vis-à-vis strengthening health systems resilience (figure 1). However, the reality and practice of these findings are left to be seen and this study has not comprehensively addressed questions about which actions may be more or less effective in ensuring resilience under varying contextual conditions. This is an area for further research, along with an expanded examination of lessons to be taken from the study countries’ differing experiences of responding to health systems shocks. TB systems have also yet to fully leverage COVID-19 pandemic innovations.^47^ Working on cross-cutting and health systems-related topics is not natural to disease programmes and requires innovation, insights from other disciplines and entrepreneurial initiatives. On top of a burnt-out health workforce, with decreasing funding and support, such a demand will be a substantial challenge.

The varying approaches to private sector engagement during COVID-19, as described by participants, appear to reflect and build on pre-pandemic public–private collaboration patterns in each country. India’s use of PPSAs to implement joint TB care strategies during COVID-19 expanded on its established PPM initiatives, which had already demonstrated success in increasing private sector TB notifications from 0% to 21% between 2012–2017.^14 15^ Indonesia’s reliance on professional associations aligned with its historically fragmented but professionally organised private healthcare system, which includes extensive faith-based hospital networks. Meanwhile, Nigeria’s focus on public–private referral policies during COVID-19 represented an attempt to address its pre-pandemic challenges with private sector engagement, where notifications had declined from 15% to 4% despite significant Global Fund support. These findings suggest that countries’ pandemic responses were shaped by their existing private sector engagement infrastructure, with more established PPM mechanisms potentially facilitating more comprehensive COVID-19 adaptation strategies.

### Strengths and limitations

A strength of this study was that it synthesised and examined insights from the COVID-19 and TB response across three countries, as reported directly by key policy-makers involved in these responses. Our study highlighted the importance of policy-makers focusing on collaborative governance, innovative financing, workforce reallocation and leveraging technology to enhance service delivery for pandemic preparedness, as well as insights from successful strategies in their contexts to improve patient–provider support and private provider engagement. However, this did not include the perspectives of individuals and providers involved more ‘downstream’ in public health and primary care (both public and private) provision. These individuals may have offered distinct insights with respect to service delivery and pandemic adaptations. Further research is needed that retrospectively examines the COVID-19 response as pertaining to TB services from more diverse perspectives and analyses the evolution of this response—and lessons learnt—following this study’s data collection time point of 2021. Further research is also needed that examines the cost-effectiveness of infection control measures, analyses TB staffing needs and constraints amid pandemic response and explores the possibilities of strengthening lab networks to support both outbreak control and service delivery.

Overall, this study contributes to understanding the intricate interplay of PPM health systems between the COVID-19 pandemic and TB care in three high-burden countries: India, Indonesia and Nigeria. The WHO Global TB Reports for 2021 and 2022 underscore the challenges in accessing TB care during the pandemic, marked by initial global TB case notification reductions followed by subsequent increases.^7 48^ Notably, the three countries exhibited distinct trends in TB notifications, emphasising the dynamic nature of pandemic responses within each context. This study underscores the importance of a collaborative, community-engaged approach as we strive for more effective and adaptable TB care in the face of future health crises and contributes to our understanding of how the resilience of both TB systems and the broader health systems of which they are a part might be strengthened.

## Conclusions

Reflecting the global UNHLM political declaration and the WHO guidance in the TB-MAF, our interviews with key policy-makers highlighted the crucial role of intersectoral collaboration, effective governance, innovative financing strategies, health workforce reallocation and technological advancements in strengthening TB service delivery amid pandemics. As we navigate the complex landscape of TB care, intersecting with the challenges posed by COVID-19, this study offers insight into the impact of the COVID-19 pandemic on private sector TB service delivery in three diverse, high-burden countries and possibilities for enhancing health systems resilience and pandemic preparedness in the realm of TB services. It will be key to continue documenting the implementation of such approaches to guide national health systems and TB programmes with concrete and practical steps in achieving resilient systems that are prepared for future pandemics.

## supplementary material

10.1136/bmjgh-2024-016180 online supplemental file 1

10.1136/bmjgh-2024-016180 Uncited online supplemental file 2

## Data Availability

All data relevant to the study are included in the article or uploaded as supplementary information.
