# Supplementary material for: Health and tuberculosis systems resilience, the role of the private sector and pandemic preparedness: insights from a cross-country qualitative study with policy-makers in India, Indonesia and Nigeria
Source: BMJ Glob Health. 2025 Jan 20;10(1):e016180. doi: 10.1136/bmjgh-2024-016180 (PMC11751827; doi:10.1136/bmjgh-2024-016180)
Supplement: Uncited online supplemental file 2 [file bmjgh-10-1-s002.docx]

# Author Reflexivity Statement

1. **How does this study address local research and policy priorities?**

India, Indonesia and Nigeria are high TB-burden countries with a combined estimated 4.4 million incident cases in 2022. This represented 40% of the total global cases. The COVID-19 pandemic had a devastating impact on global TB case notifications, with a 20% drop in 2020, with significant rebound in 2022. Globally, the reporting of TB cases from the private sector does not match the proportion of individuals who first seek healthcare from these providers. Increasing TB notifications from the private sector and identifying cross-cutting factors impacting pandemic preparedness are priorities for the national governments of high-burden countries.

1. **How were local researchers involved in the study design?**

This research endeavour was co-designed by COO, LJB, and MP, in collaboration with the TB PPM Learning Network co-authors (PH, VY, AS, NAV, PT, SS, and JSK) and local researchers BOF, VY, and BWL. COO, BOF, BWL, VY, JSK, SS, and MP are natives of the three study countries.

1. **How has funding been used to support the local research team?**

The study funded local research team expenses related to the policymaker interview data collection and cleaning, staff time for research support, and expenses for disseminating results to local stakeholders.

1. **How are research staff who conducted data collection acknowledged?**

Several local research staff involved in managing the data collection and cleaning processes (VY, BOF, and BWL) are included as co-authors. All other staff and contractors involved in the field work are acknowledged.

1. **Do all members of the research partnership have access to study data?**

The data collected in this study was collected, cleaned, and managed by local research staff. It was transmitted to team members at the Universities of McGill and Waterloo for analysis. All members of the research team have access to these data.

1. **How was data used to develop analytical skills within the partnership?**

Data analysis was performed by LJB, COO, VY, BWL, and PH. The team experimented with different ways of representing the data and shared ideas with each other.

1. **How have research partners collaborated in interpreting study data?**

The research team worked together over several months to analyse and interpret the results from this research, with frequent inputs from more senior academics. This process was crucial in ensuring that the interpretation and contextualizing of the findings were true to the local context.

1. **How were research partners supported to develop writing skills?**

The research team responsible for analysing, interpreting, and writing this report were predominantly early career researchers, including research assistants and a postdoctoral fellow. All authors made contributions to the manuscript.

1. **How will research products be shared to address local needs?**

The results from this study have been disseminated to local stakeholders including the National TB Programs and TB program managers. We have also shared the results with the TB PPM Learning Network through a webinar and to the WHO TB PPM Working Group. Additionally, this manuscript will be published as open access.

1. **How is the leadership, contribution and ownership of this work by LMIC researchers recognized within the authorship?**

Co-authors VY, BOF, and BWL are LMIC researchers. Local staff within the research teams are acknowledged.

1. **How have early career researchers across the partnership been included within the authorship team?**

The vast majority of co-authors are early career researchers, including from LMICs (LJB, COO, VY, BWL, NAV, AS, BOF, PT, JSK, and SS).

1. **How has gender balance been addressed within the authorship?**

Nine authors are female, and three authors are male.

1. **How has the project contributed to improvements in local infrastructure?**

This project has not directly contributed to improvements in local infrastructure.

1. **What safeguarding procedures were used to protect local study participants and researchers?**

All field staff were trained and study instruments were pilot-tested to ensure high data quality. Collected data were anonymized by field staff before being shared with the rest of the research team. To protect patients and field staff from potential COVID-19 exposure during data collection, several precautionary measures were put in place. During training, temperature screening was carried out on the personnel before admittance into the training hall. The wearing of facemasks was enforced throughout the training period. Also, alcohol-based hand sanitizers were made available to the participants.
